# Fatty acid metabolism shapes immune responses in chronic lymphocytic leukemia

**DOI:** 10.1186/s40364-025-00753-7

**Published:** 2025-03-12

**Authors:** Yang Zhang, Jun Ma, Peipei Li, Kang Lu, Yang Han, Xinting Hu, Xiaosheng Fang, Xin Wang, Ya Zhang

**Affiliations:** Department of Hematology, Shandong Provincial Hospital Affiliated to Shandong First Medical University, No.324, Jingwu Road, Jinan, Shandong 250021 China

**Keywords:** Chronic lymphocytic leukemia, Fatty acid metabolism, Immunophenotypes, PI3K inhibitors

## Abstract

**Background:**

Fatty acids serve as a crucial energy source for tumor cells during the progression of chronic lymphocytic leukemia (CLL). The present study aims to elucidate the characteristics of fatty acid metabolism (FAM) in CLL, construct a related prognostic score, and investigate the regulatory role and mechanisms of FAM in CLL development.

**Methods:**

Bulk RNA sequencing data from CLL patients and healthy controls were analyzed to identify differentially expressed fatty acid metabolic genes. *FAM-score* was constructed using Cox-LASSO regression and validated. Single-cell RNA sequencing was used to analyze the expression of key FAM genes in CLL immune cell subsets and investigate cellular communication. Functional assays, including cell viability, drug sensitivity, and oxygen consumption assays, were performed to assess the impact of fatty acid oxidation (FAO) inhibition on CLL cells.

**Results:**

Three FAM-related genes (LPL, SOCS3, CNR1) were identified with independent prognostic significance to construct the risk score. The *FAM-score* demonstrated superior prognostic performance compared to the Binet stage and was associated with established clinical prognostic markers. Single-cell analysis revealed distinct expression patterns of LPL, SOCS3, and CNR1 across CLL immune cell subsets. Cellular communication analysis highlighted the regulatory role of distinct B cell and Treg subsets in the CLL microenvironment. CLL patients with high *FAM-score* displayed distinct immune infiltration patterns, with increased FAO pathway activity. Inhibition of FAO reduced CLL cell viability, synergistically enhanced the efficacy of the PI3K inhibitor idelalisib.

**Conclusion:**

The present study constructed a prognostic risk score based on FAM gene expression, revealing related immune phenotypic differences and exploring the regulatory role of FAO in CLL development. Targeting fatty acid metabolism potentially modulates the CLL immune microenvironment and synergistically enhances the efficacy of PI3K inhibitors.

**Supplementary Information:**

The online version contains supplementary material available at 10.1186/s40364-025-00753-7.

## Introduction

Chronic lymphocytic leukemia (CLL) is a relatively indolent hematologic malignancy of mature CD19^+^CD5^+^ B cells. CLL cells exhibit increased dependence on free fatty acid oxidation (FAO) for energy compared to normal B cells [1]. Fatty acid metabolism has implications for drug resistance in CLL, with ibrutinib resistance linked to FAO and abnormal phospholipid levels associated with chemotherapy resistance [2–5]. However, the gene expression patterns related to fatty acid metabolism in CLL have not been systematically studied.

Extensive research has shown that metabolism reprograming, especially fatty acid metabolism (FAM), shapes an immune suppressive microenvironment that promotes tumor progression [6–8]. Multi-omics analysis in colorectal cancer patients has highlighted the impact of lipid metabolism on immunogenicity [6]. Correspondingly, the fatty acid transport pathway is directly involved in the pathological activation of neutrophils in a variety of tumors such as lymphoma, lung cancer, and pancreatic cancer [7]. Moreover, tumor cells and CD8^+^ T cells showed different metabolic adaptability to obesity in a mouse cell model of colorectal cancer, resulting in changes in fatty acid distribution in high-fat diet tumors, thereby impairing the infiltration and function of CD8^+^ T cells [8]. As a lymphoproliferative disease with the perturbation of the immune system, the pathogenesis and progression of CLL are highly dependent on the immune microenvironment. Nevertheless, it has not been entirely elucidated whether alterations in fatty acid metabolism in CLL perturb immune cell fate and function.

In this study, we integrated bulk and single-cell transcriptomic data from CLL patients to comprehensively characterize the role of FAM in CLL. Based on the FAM-specific transcriptomic signature, we developed a prognostic risk model *FAM-score* containing 3 genes (LPL, SOCS3, and CNR1). Mechanistically, these genes are mainly related to the FAO and lipogenesis [9–11]. Moreover, the relationship between the CLL immunophenotype and hub genes related to FAM was investigated combined with single-cell transcriptome analysis of CLL patients. Meanwhile, this study found that the inhibition of FAO impeded the survival of CLL cells and enhanced their sensitivity to PI3K inhibitor idelalisib, paving the way for the modulation of fatty acid metabolism to improve CLL therapy.

## Methods

### Patient specimens

The study was approved by the Medical Ethics Committee of Shandong Provincial Hospital, and each patient gave the informed consent. CLL was diagnosed according to the updated International Workshop on Chronic Lymphocytic Leukemia (IWCLL) criteria [12]. CLL primary cells were extracted and isolated from the peripheral blood of 5 first-treatment CLL patients from Shandong Provincial Hospital’s Department of Hematology as previously published [13, 14]. In addition, peripheral blood mononuclear cells from 5 primary CLL patients and 2 healthy controls were processed for 10x Genomics single-cell RNA sequencing (scRNA-seq).

### Data acquisition

The fatty acid metabolism-related genes were defined as genes enriched in fatty acid metabolic pathways based on the Molecular Signatures Database (MSigDB, https://www.gsea-msigdb.org/gsea/msigdb). Bulk and single-cell transcriptome data of CLL patients were obtained from the Gene Expression Omnibus database (GEO; https://www.ncbi.nlm.nih.gov/geo/) and International Cancer Genome Consortium (ICGC; https://dcc.icgc.org/). To establish and validate the *FAM-score*, the RNA-seq data, scRNA-seq data and clinical prognosis information of 791 CLL patients from multiple cohorts were incorporated.

### Construction and validation of the risk score model base on the fatty acid metabolic genes

The differentially expressed genes (DEGs) with significant differences in expression levels between CLL and normal samples were calculated using DESeq2 [15]. The characteristic genes of fatty acid metabolism in CLL are defined as the intersection of DEGs and the set of fatty acid metabolism-related genes. Statistical significance was assigned to genes with a |logFC| of more than 1.2.

The Cox-Least absolute shrinkage and selection operator (LASSO) regression model was performed in ICGC cohort. The risk score was calculated as: $\:Risk\:Score=\:{\sum\:}_{1}^{i}(Coefi\:\times\:ExpGenei)$. The “Coef” represents regression coefficients, and “ExpGene” is the expression values of genes from the prognostic risk score model. Finally, the reliability and applicability of the prognostic risk score model were further validated by Kaplan-Meier analysis and time-dependent ROC curve in the external validation set. For a deeper understanding of the relationship between the risk score and clinical features, clinical correlation analysis was conducted among patients in different subgroups. CLL patients in the ICGC cohort were categorized by age, sex, Binet stage, IGHV mutation status, TP53 mutation status and disease status at the end of follow-up.

### Expression signatures and metabolic activity in single-cell transcriptomes

For visualization purposes, dimensional reduction was carried out using tSNE. The analysis of intercellular communication was performed with CellChat. Furthermore, the activity of fatty acid metabolic pathways in each single cell sample was calculated by UCell [16]. The knnDREMI (conditional-Density Resampled Estimate of Mutual Information) was applied to estimate the functional relationship of hub genes expression to other genes across the dynamic range of expression [17]. 

### Metabolic and immune function enrichment analysis associated with *FAM-score*

The fatty acid metabolism pathways activity was assessed by single-sample gene set enrichment analysis (ssGSEA). Subsequently, the CIBERSORT deconvolution algorithm was employed to analyze the cellular composition of the samples based on gene expression profiles [18]. Moreover, Gene Ontology (GO) and Kyoto Encyclopedia of Genes and Genomes (KEGG) analysis were conducted to decipher the primarily enriched signaling pathways and biological functions between the high- and low-risk groups in the training set [19]. 

### Cell metabolism assay

The measurement of extracellular oxygen consumption was conducted using the Extracellular Oxygen Consumption Assay Kit (Abcam, Cambridge, England). Following the manufacturer’s instructions, the Fatty Acid Oxidation Assay Kit (Abcam, Cambridge, England) and the Extracellular Oxygen Consumption Assay Kit were used to measure fatty acid oxidation. The initial slope of fluorescence intensity was calculated from the same linear portion of the curve (0–10 min) in the Fatty Acid Oxidation assay using GraphPad Prism.

### RNA isolation and quantitative real-time PCR

Total RNA was purified with SPARKeasy cell RNA kit (SparkJade, Jinan, China) and reversed transcribed into cDNA using a reverse transcription kit (Agbio, Changsha, China). Real-time PCR was performed in a Light Cycler 480 II Real-Time PCR system (Roche Diagnostics, Basel, Switzerland) using SYBR Green (Agbio, Changsha, China). Real-time PCR of each sample was performed in triplicate. Results were obtained using the sequence detection software Light cycler 480 and analyzed using GraphPad Prism version 9.0 statistical software.

### Analysis of cell proliferation and apoptosis

1 × 10^4^ cells were placed into each well of a 96-well culture plate and treated with the relevant drug doses. Following incubation periods of 24, 48, and 72 h, 10 μl of CCK-8 (DOJINDO, Kumamoto, Japan) solution was added to each well. The contents were mixed thoroughly, and absorbance was measured at 450 nm after 2 h.

1 × 10^6^ cells were inoculated into each well of a six-well plate and treated with the appropriate drug doses. After 24 h, the cells were collected and washed with 1×PBS. For apoptosis analysis, the cells were resuspended in 100 μL of binding buffer, and 5 μL of 7-AAD and Annexin V-PE were added to each tube. The samples were incubated at room temperature in the dark for 15 min, followed by analysis using the ATTUNE NXT Flow Cytometer (Thermo Fisher, Massachusetts, USA).

### Statistical analysis

All statistical analyses were accomplished via R software (Version 4.2.3, http://www.R-project.org). The comparison of each Kaplan-Meier curve contained in this study was completed by the log-rank test. The differences of the immune infiltrates in the different subgroups and *FAM-score* in various clinicopathologic parameters were detected by the Wilcoxon test. Univariate and multivariate Cox regression analyses were exploited to screen out the OS-related hub genes and the independent prognostic indicators of OS. Statistical significance was determined as *p* < 0.05.

## Results

### Dissecting FAM-related genes expression in CLL by integrating bulk and single-cell transcriptomics

Differential expression analysis of CLL cells and healthy B cells identified genes with altered expression, particularly those related to FAM, based on the hypothesis that FAM dysregulation contributes to CLL. To identify potential prognostic markers or therapeutic targets by pinpointing metabolic vulnerabilities in CLL, differential expression analysis identified 34 DEGs associated to fatty acid metabolism in expression arrays of 180 CLL blood samples and 32 sorted CD19^+^ B cell samples from healthy donors in GSE50006 (Figure S1A). To investigate the prognostic value of these altered metabolic genes, 34 FAM-related DEGs were analyzed in the ICGC cohort (training set) using univariate Cox regression, identifying 10 prognostic genes (*p* < 0.05; Figure S1B). LASSO-Cox regression analysis was then performed to narrow the number of genes. Finally, 3 hub genes (LPL, SOCS3, CNR1) with independent prognostic significance were selected to construct the robust risk score named *FAM-score* (Figure S1C-F).

**Fig. 1:**
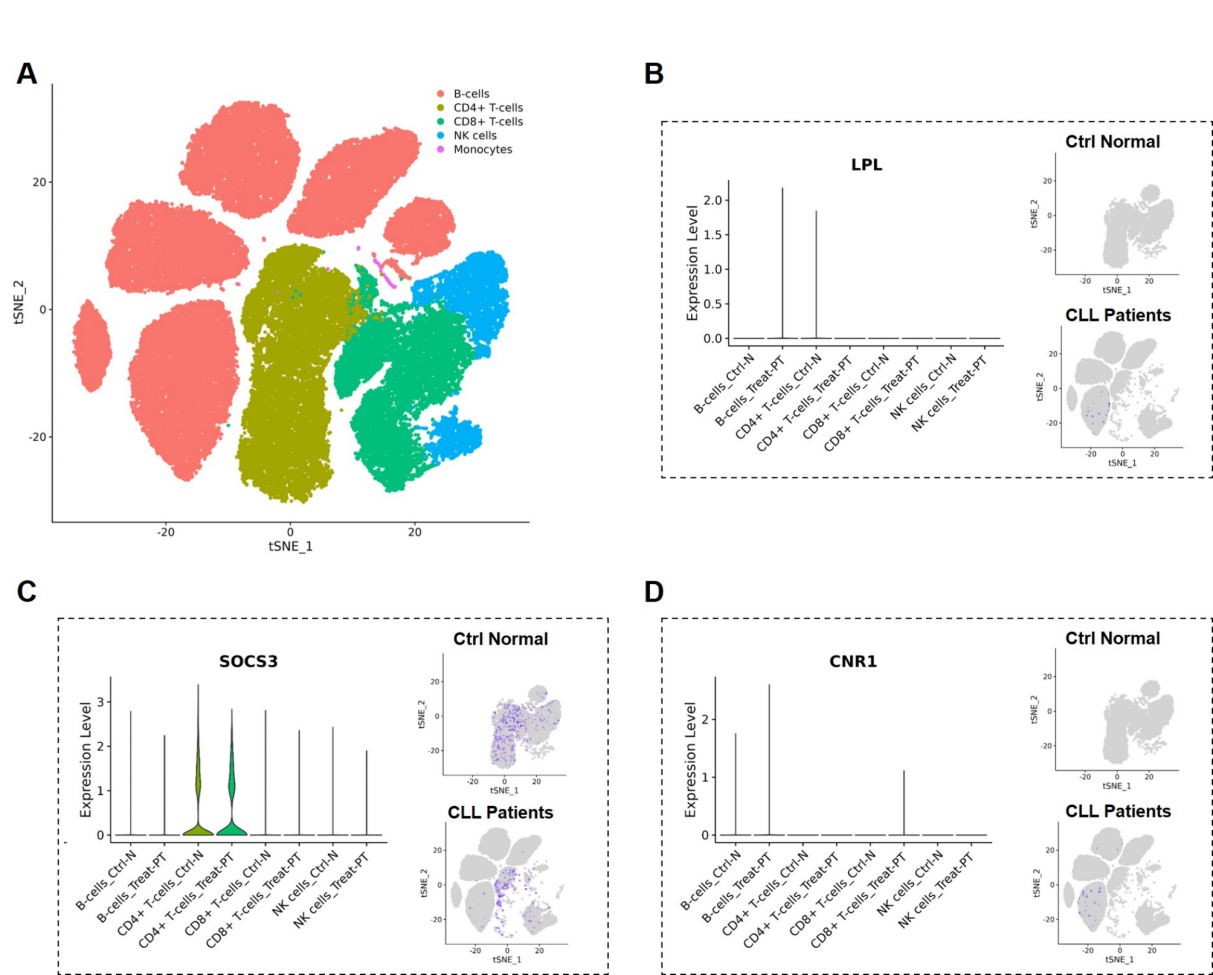
Expression characteristics of FAM at the single-cell level. (**A**) The expression of these hub genes LPL, SOCS3, and CNR1 in CLL patients and healthy controls. (**B**) The expression characteristics of LPL. (**C**) The expression characteristics of SOCS3. (**D**) The expression characteristics of CNR1

To further explore the role of FAM in CLL immune regulation, the expression characteristics of LPL, SOCS3, and CNR1 at the single-cell level were analyzed. First, we compared the expression of these hub genes in CLL patients and healthy controls (Fig. 1A). In healthy control samples, LPL is predominantly expressed on CD4^+^ T cells, whereas in CLL patients, LPL is expressed at relatively higher levels on B cells (Fig. 1B). SOCS3 tends to be expressed on CD4^+^ T cells, but its expression levels in CLL patients are lower than in healthy controls (Fig. 1C). CNR1, on the other hand, exhibits exceedingly low expression, with only minimal presence observed in B cells. However, CNR1 is also slightly expressed in CD8^+^ T cells in CLL patients (Fig. 1D). Furthermore, the expression signature of hub genes in CLL samples at different time points supports these findings (Figure S1G-J). The t-SNE profiles revealed cell subpopulation specificity in the expression of key genes in CLL, suggesting that fatty acid metabolism potentially induces differences between immune cell types.

### Alterations in FAM induce immune dysfunction in CLL patients

**Fig. 2:**
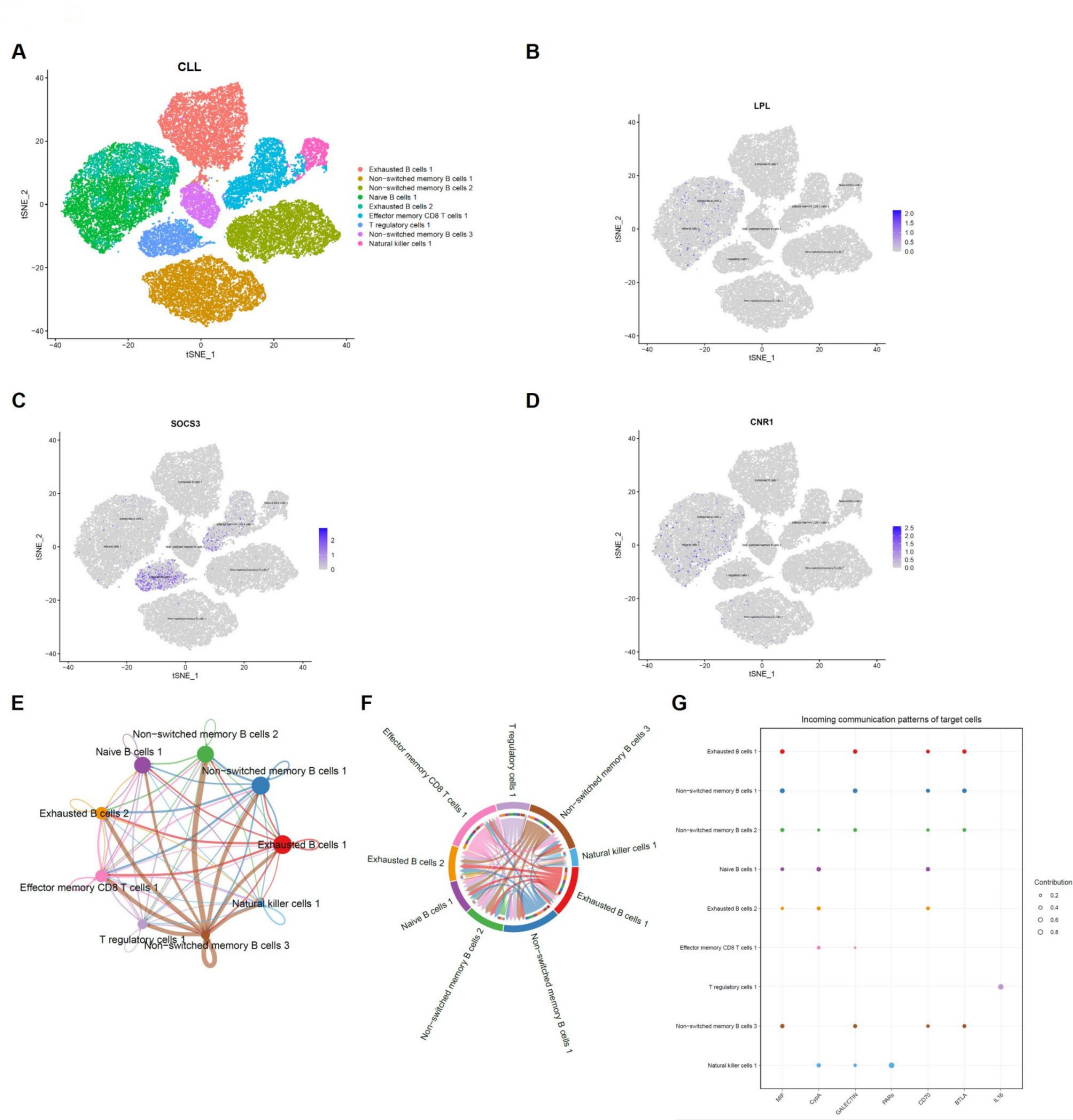
Expression characteristics of FAM genes in immune cell subsets. (**A**) t-SNE visualization of immune cell clusters from CLL patients. (**B**-**D**) The expression characteristics of LPL, SOCS3, and CNR1 in immune cell clusters from CLL patients. (**E**-**F**) The interaction relationship between immune cells in CLL patients. The thickness of the connecting line indicates the strength of the interaction. (**G**) Incoming communication patterns of immune cells in CLL patients

To determine the function and mechanism of FAM-related genes in the immune microenvironment of CLL patients, single-cell transcriptome data from CLL patients were further subdivided into different immune cell clusters (Fig. 2A). The expression characteristics of LPL, CNR1, and SOCS3 were then investigated within these clusters (Fig. 2B-D). Cellular communication analysis revealed that exhausted B cells, regulatory T cells (Tregs), and a population of unswitched memory B cells exert broad regulatory influence within the CLL immune microenvironment (Fig. 2E-G). Notably, SOCS3 expression was highly concentrated within the Treg population and, furthermore, within the effector memory CD8^+^ T cell population targeted by these Tregs. This localized SOCS3 expression underscores its crucial role in modulating immune responses within these specific immune cell subsets in CLL patients.

These findings are corroborated by bulk RNA sequencing data in the ICGC cohort, which revealed distinct immune infiltration patterns between the two *FAM-score* subgroups (Figure S2B-C). High *FAM-score* CLL patients exhibited significantly decreased levels of activated CD4^+^ T cells, CD8^+^ T cells, γδ T cells, and dendritic cells, concurrent with an expansion of M2 macrophages, plasma cells, follicular helper T cells, and activated NK cells. These findings demonstrate that CLL patients exhibit two distinct immune phenotypes based on differential expression of genes related to fatty acid metabolism.

Mechanistically, GO enrichment analysis of the bulk RNA-seq data linked the *FAM-score* to pathways involved in cell fate determination and specification, foam cell differentiation, and unsaturated fatty acid metabolism (Figure S2D). Furthermore, KEGG analysis showed enrichment of immune cell differentiation, activation, and immune response regulation pathways in the high-risk subgroup (Figure S2E). These alterations in fatty acid metabolism pathways may thus mediate immune cell differentiation and function, contributing to CLL development and progression.

### Enhanced fatty acid oxidation drives CLL progression through the immune regulation

**Fig. 3:**
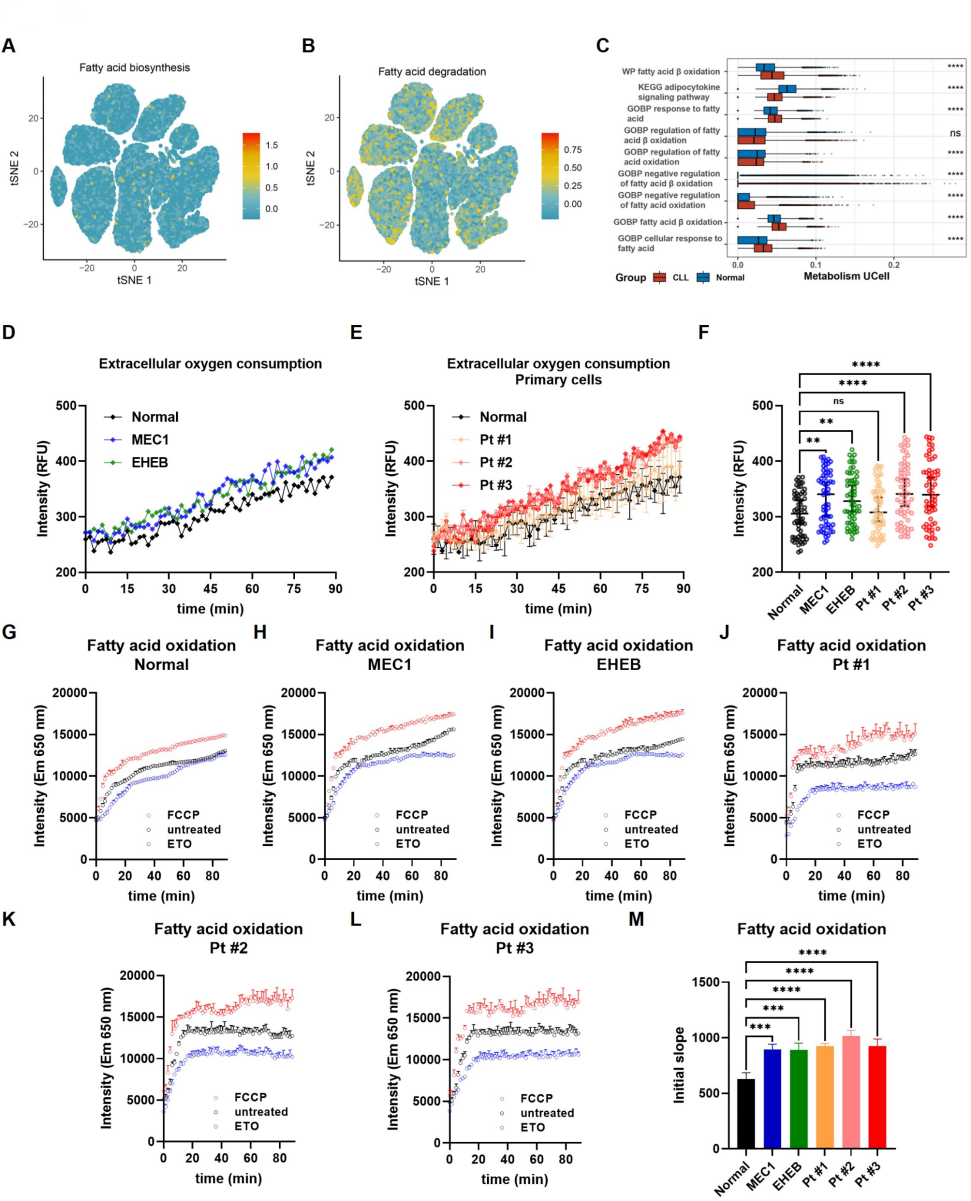
CLL fatty acid metabolism is characterized by increased fatty acid oxidation. (**A**-**B**) Activity of fatty acid biosynthesis and degradation pathways at the single-cell level. (**C**) Comparison of the activities of major fatty acid metabolic pathways in CLL patients (CLL) and healthy controls (Normal). (**D**) Extracellular oxygen consumption assay in MEC1 cells, EHEB cells, and normal peripheral blood mononuclear cells. (**E**) Extracellular oxygen consumption assay in CLL primary cells, and normal peripheral blood mononuclear cells. (**F**) Comparison of extracellular oxygen consumption levels between CLL cells and normal peripheral blood monocytes. (**G**-**L**) Fatty acid oxidation assay in CLL cells and normal peripheral blood mononuclear cells. (**M**) Comparison of fatty acid oxidation activity between CLL cells and normal peripheral blood monocytes. NS: not significant; *: *p* < 0.05; **: *p* < 0.01; ***: *p* < 0.001

While fatty acid metabolic reprogramming is known to influence tumor progression in various cancers by shaping an immunosuppressive microenvironment, the specific impact of altered fatty acid metabolism on immune cell fate and function in CLL remained unclear [6–8]. Fatty acid metabolism encompasses the breakdown of fatty acids for energy production and their synthesis for storage or molecular building blocks. To explore how fatty acid metabolism modulates the CLL immune phenotype, the scMetabolism algorithm was applied to quantify fatty acid metabolic activity from single-cell transcriptomes. This analysis revealed that degradation pathways were more active than biosynthesis pathways in CLL cells (Fig. 3A-B). Furthermore, fatty acid β-oxidation and cellular response to fatty acids pathways were significantly more active in CLL patients than in healthy controls (Fig. 3C). To identify alterations of fatty acid metabolism in CLL patients with elevated *FAM-scores*, ssGSEA analysis of the FAM-related pathways was implemented on the ICGC cohort (Table S1). Consistent with previous studies, multiple pathways related to fatty acid oxidation, synthesis, and transport showed significantly elevated transcriptional activities in the high-risk subgroup (Figure S2A). To validate our transcriptomic findings, we measured extracellular oxygen consumption and fatty acid oxidation in peripheral blood mononuclear cells from CLL cell lines, primary CLL patient samples, and healthy controls (Fig. 3C-D, F-J). CLL cells exhibited significantly higher extracellular oxygen consumption and fatty acid oxidation compared to healthy controls (Fig. 3E, K).

**Fig. 4:**
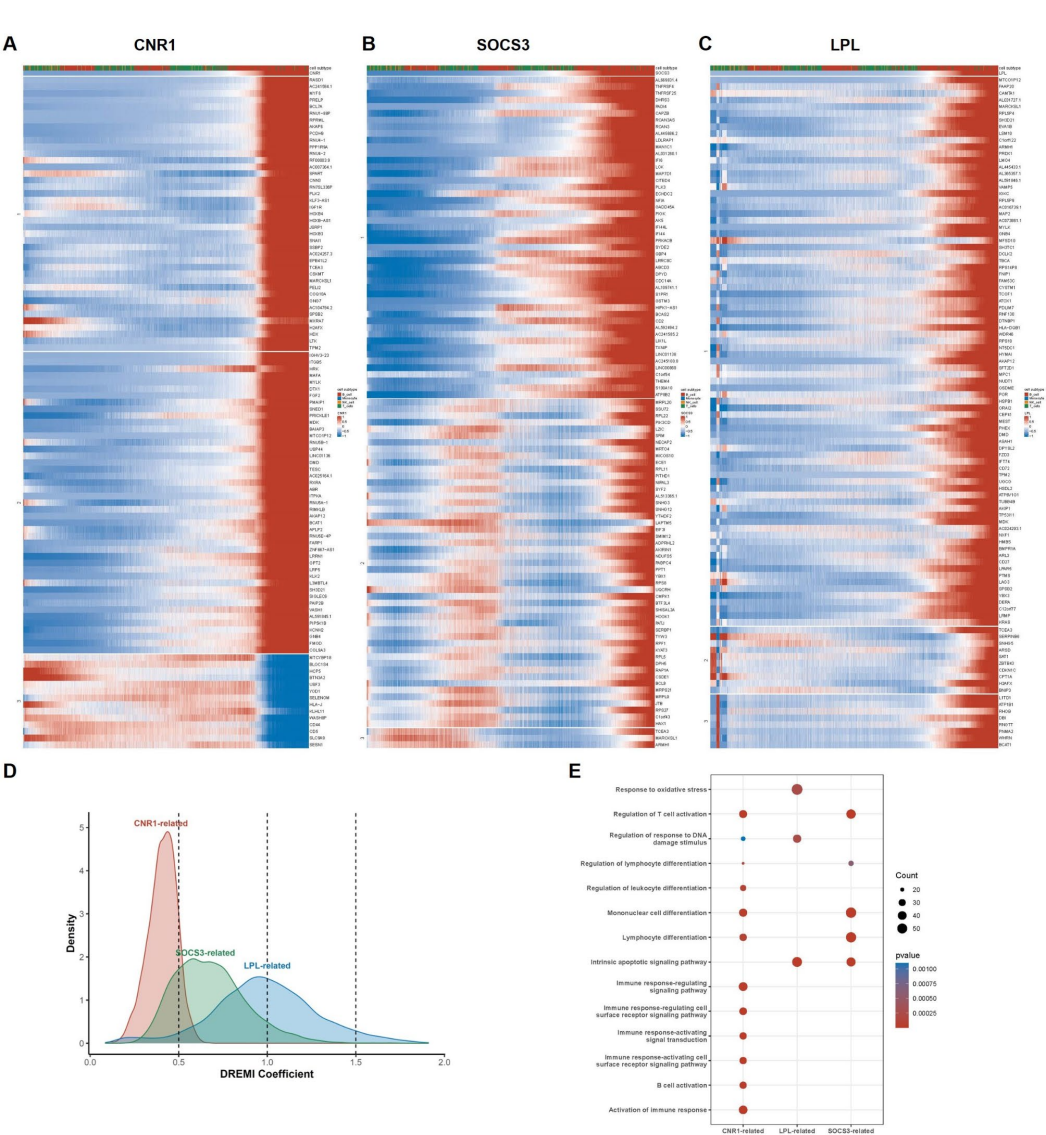
Identification of interacting molecules of FAM-related genes in the CLL single-cell transcriptome. (**A**) Heatmap of expression of CNR1-interacting molecules in CLL single-cell transcriptome. (**B**) Heatmap of expression of SOCS3-interacting molecules in CLL single-cell transcriptome. (**C**) Heatmap of expression of LPL-interacting molecules in CLL single-cell transcriptome. (**D**) The distribution of gene DREMI coefficient related to CNR1, LPL and SOCS3. (**E**) KEGG pathway enrichment analysis of FAM-related gene interactors

Subsequently, the reciprocal molecules of the key genes were identified in the single-cell transcriptome to investigate the mechanism of FAM-induced differences of CLL immunophenotype (Fig. 4A-C). The distribution of gene DREMI coefficient related to CNR1, LPL and SOCS3 were shown in Fig. 4D, and the thresholds for interacting genes are > 0.5, > 1 and > 1.5 respectively. Consistent with the results of functional enrichment analysis in bulk RNA-sequencing data, the interacting genes of CNR1, LPL, and SOCS3 are all enriched in the immune cell activation pathway and its regulatory pathways (Fig. 4E). These results suggest that within the context of fatty acid metabolism reprogramming, immune regulation might serve as a potential mechanism to promotes the progression of CLL.

### Inhibition of fatty acid oxidation in CLL cells impedes survival and proliferation

**Fig. 5:**
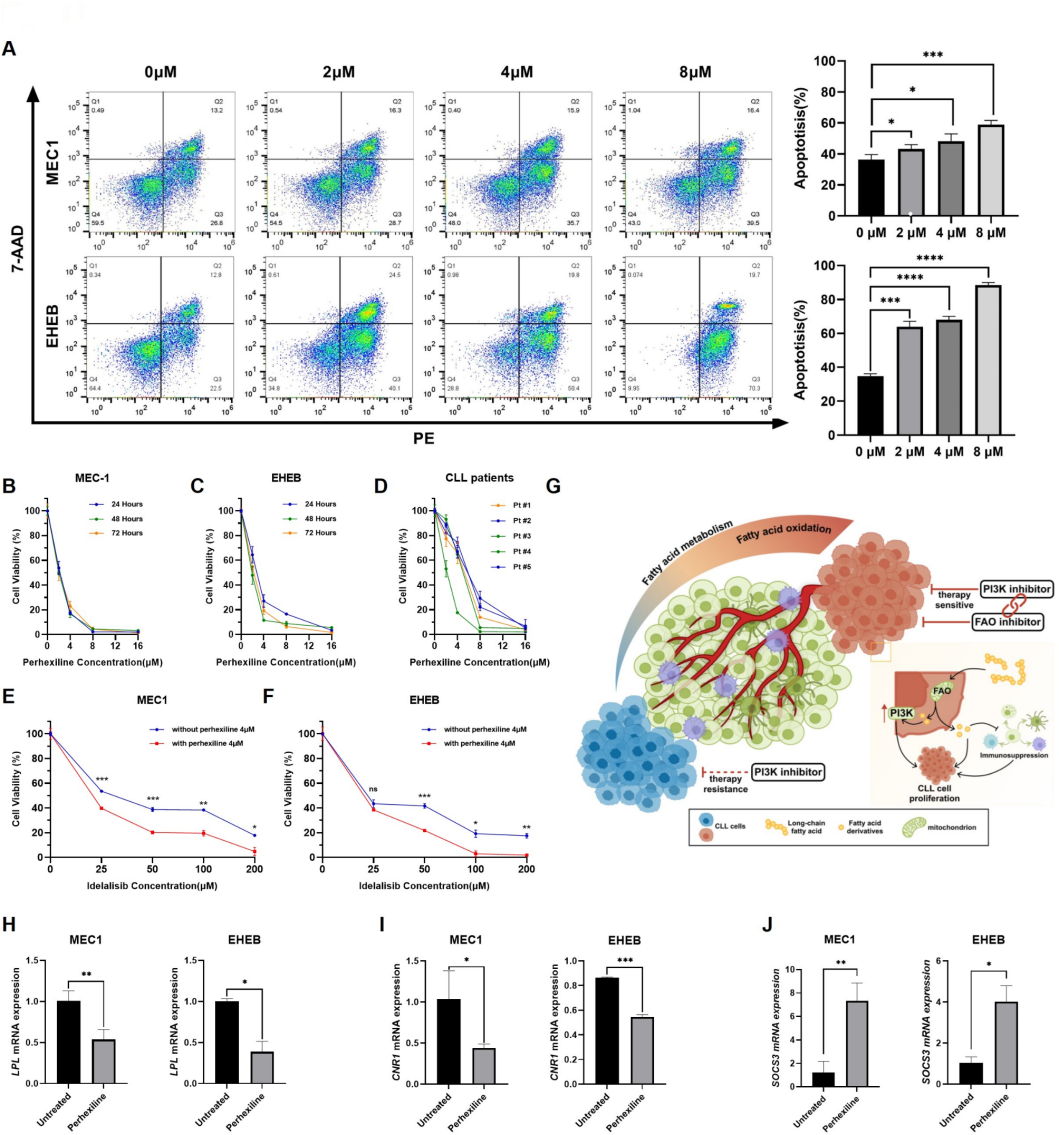
Fatty acid oxidation inhibition impeded CLL cells and enhanced sensitivity to PI3K inhibitors. (**A**-**C**) Cell proliferation analysis of MEC1, EHEB cells and primary cells from CLL patients treated with perhexiline. (**D**) Mechanisms of fatty acid metabolism regulating the development of CLL. (**E**-**F**) Sensitivity assay for the PI3K inhibitor idelalisib in MEC1 and EHEB cells treated with perhexiline. (**G**) Apoptosis analysis of MEC1 and EHEB cells treated with FAO inhibitors perhexiline. Symbols on the top represent significant difference. (**H**) The relative transcription levels of hub genes in MEC1 and EHEB cells treated with FAO inhibitor perhexiline at a concentration of 4 μ M for 24 h. NS: not significant; *: *p* < 0.05; **: *p* < 0.01; ***: *p* < 0.001; ****: *p* < 0.0001

Both bulk and single-cell RNA sequencing analyses demonstrated significant upregulation of FAO pathways in high-risk CLL patients. To further investigate the effects of targeted regulation of fatty acid metabolism on CLL cells, we inhibited FAO in CLL cells by blocking fatty acid entry into mitochondria using perhexiline, according to published literature [20, 21]. The MEC1, EHEB, and primary CLL cells co-cultured with perhexiline (0 μM, 2 μM, 4 μM, and 8 μM) exhibited a concentration-dependent decrease in viability across various durations, as shown by cell viability assays (Fig. 5A). Furthermore, co-culturing MEC1 and EHEB cells with the PI3K inhibitor idelalisib in the presence or absence of 4 μM perhexiline revealed an enhanced sensitivity of CLL cells to idelalisib upon FAO inhibition (Fig. 5B-F). Flow cytometric analysis confirmed a significant increase in apoptosis in CLL cells following FAO inhibition (Fig. 5G). In line with our results from integrated bulk and single-cell RNA analyses, treating CLL cells with Perhexiline to inhibit FAO revealed a downregulation of LPL and CNR1 gene expression compared to untreated controls (Fig. 5H-I). Conversely, a significant upregulation of SOCS3 expression was observed (Fig. 5J).

Furthermore, analysis of the *FAM-score*’s relationship with clinical features in the ICGC cohort revealed significant differences across subgroups, with the high-risk group associated with age > 60, male gender, unmutated IGHV, advanced Binet stage, and disease progression (*p* < 0.05; Figure S3A-E). Further analysis of cytogenetic and transcriptomic data from 109 CLL patients showed lower *FAM-score* (*p* < 0.05; Figure S3F-G). Kaplan-Meier analysis confirmed the *FAM-score*’s prognostic value across different sexes, ages, and IGHV mutation statuses (*p* < 0.05; Figure S4A-F), though significance was observed only in TP53 wild-type patients, possibly due to limited TP53-mutated samples (Figure S4G-H). These findings suggest that FAM-related genes, acting through the fatty acid oxidation pathway, play a crucial role in CLL cell survival and proliferation. Furthermore, inhibiting fatty acid oxidation synergistically enhances the efficacy of PI3K inhibitor in CLL.

## Discussion

The present study identified a novel prognostic risk score based on fatty acid metabolic genes and has elucidated the immunophenotypic differences in CLL related to the FAM-related genes. Integrated analysis of transcriptomic data and cellular assays demonstrates that increased fatty acid oxidation is a prominent feature of CLL metabolic reprogramming. Furthermore, pharmacological inhibition of FAO, the most significantly altered FAM pathway in the high-risk CLL subgroup, enhances CLL cell sensitivity to PI3K inhibitors. Collectively, these findings demonstrate that the observed reprogramming of fatty acid metabolism, specifically the increased FAO pathway in CLL cells, drives their immunosuppressive phenotype and resistance to PI3K inhibitors, thereby promoting the development of CLL (Fig. 5D).

LPL, CNR1, and SOCS3 were identified as key regulators of fatty acid metabolism and used to construct the *FAM-score*, whose expression levels exhibit significant differences between samples of CLL and healthy controls. LPL is aberrantly overexpressed in chronic lymphocytic leukemia cells, and facilitates the use of fatty acids for energy production in CLL cells, contributing to their survival and proliferation [4, 22–24]. It is regulated by B-cell receptor signaling and STAT3, and its expression is associated with adverse prognosis [2, 25, 26]. SOCS3 regulates FAO and energy metabolism through the leptin signaling pathway [11, 27, 28]. In CLL, the expression of the SOCS3 is suppressed and significantly associated with leukemia progression [29, 30]. CNR1, which encodes cannabinoid receptor 1, plays a significant role in regulating lipid metabolism by mediating inflammatory responses and oxidative stress through the NF-κB signaling pathway [31–33]. In CLL, CNR1 is overexpressed compared to normal B cells and is associated with adverse prognosis [34–36]. These genes are directly or indirectly implicated in FAO, which is consistent with our findings. In comparison with healthy controls, the FAO pathway demonstrates heightened activity in patients diagnosed with CLL, particularly within high-risk subgroups, suggesting that the reprogramming of fatty acid metabolism might change the survival of CLL patients.

The *FAM-score* consistently predicts clinical outcome in diverse CLL patient profiles, except for cases involving TP53 mutations. Existing studies have highlighted that TP53 mutations mediate the disorder of fatty acid metabolism and participate in the lipolysis reaction of adipocytes [37–39]. However, due to the limited number of TP53 mutation cases (only 16 cases) in this study, we cannot definitively conclude the ineffectiveness of *FAM-score* in CLL patients with TP53 mutations. Therefore, further studies are needed to clarify the relationship between FAM reprogramming and TP53 mutations in CLL patients.

The importance of crosstalk between the spectrum of immune cells and CLL cells in the tumor microenvironment has been highlighted in previous studies [40]. The present study revealed that expression signatures of genes involved in fatty acid metabolism effectively manipulate the immunophenotype of CLL patients. Earlier work have demonstrated the impact of metabolic factors on immune cell differentiation and function [8, 41–46]. Consistent with these findings, the present study highlights distinct immune cell subsets affected by FAM, particularly FAO, further supporting the opinion that fatty acid metabolism regulates immune cell differentiation. In the present study, single-cell sequencing of CLL patients revealed significant immune cell subset-specific expression of the fatty acid metabolism (FAM) genes LPL, SOCS3, and CNR1. This distinct expression pattern was further associated with the immunosuppressive phenotype observed in the high-risk patient subgroup identified through immune infiltration analysis. The abundance of DCs responsible for activating T cells was significantly reduced in CLL patients with increased *FAM-score*, and essential effector T cell subsets (such as activated CD4^+^ T cells, CD8^+^ T cells, and γδ T cells) were concomitantly reduced, collectively revealing that immunosuppressive features are associated with elevated *FAM-score*.

The PI3K signaling pathway materially influences cell proliferation, survival, and metabolism in response to external cues, abnormal activation of which is a common oncogenic event in human cancers [47–50]. PI3Kδ inhibitors, such as idelalisib, have been approved by the FDA for the treatment of relapsed chronic lymphocytic leukemia, follicular non-Hodgkin’s lymphoma, and small lymphocytic lymphoma, demonstrating their clinical significance in these hematological malignancies [47]. Positioned at the intersection of metabolism and immunity, the PI3K pathway has intriguing implications. For instance, in a mouse model of gastric cancer, PI3K inhibitors targeting the PI3K-AKT-mTOR pathway reduced free fatty acids accumulation and enhanced the efficacy of immune checkpoint (PD-1) inhibitors [42]. Similarly, using the PI3Kα inhibitor CYH33 in pancreatic cancer mouse models enhances tumor microenvironment fatty acid metabolism, activating CD8^+^ T cells and triggering anti-tumor immunity [51]. In the present study, inhibition of FAO in CLL cells effectively reinforced the antitumor effects of idelalisib. The observed synergy between these two approaches underscores the critical interplay between metabolic reprogramming, particularly FAO, and PI3K signaling in CLL, thus strongly supporting further investigation of combination therapies targeting both pathways to overcome resistance and improve treatment outcomes.

## Conclusion

In summary, the present study has identified a robust clinical prognostic score based on fatty acid metabolic genes. Despite its limitations, our study contributes to the understanding of fatty acid metabolism reprogramming in CLL. We found that CLL cells dependence on FAO regulates the immune function, which contributes to both CLL cells proliferation and the establishment of an immunosuppressive microenvironment. Additionally, inhibition of FAO in CLL cells enhances the therapeutic efficacy of PI3K inhibitor and suppresses cell proliferative activity. Our work elucidates the impact of fatty acid metabolism on CLL immunophenotype and disease progression. FAM modulation might represent a promising immunotherapeutic strategy by reversing the immunosuppressive microenvironment, while inhibition of FAO enhances PI3K inhibitor efficacy through synergistic effects.

## Electronic supplementary material

Below is the link to the electronic supplementary material.

Supplementary Material 1

**Supplementary Material 2:** Table S1

**Supplementary Material 3:** Table S2

**Supplementary Material 4:** Table S3

**Supplementary Material 5:** Table S4

## Data Availability

No datasets were generated or analysed during the current study.
