# Supplementary material for: Fatty acid metabolism shapes immune responses in chronic lymphocytic leukemia
Source: Biomark Res. 2025 Mar 12;13:42. doi: 10.1186/s40364-025-00753-7 (PMC11905569; doi:10.1186/s40364-025-00753-7)
Supplement: Supplementary file 1 — Supplementary Material 1 [file 40364_2025_753_MOESM1_ESM.pdf]

Supplementary materials

Supplementary Table legends

* The corresponding tables themselves are submitted as separate files due to their size, as permitted by the journal's guidelines.

Table S1. ssGSEA results of fatty acid metabolism pathways.

Please refer to the files named "Table S1.xlsx"

The ssGSEA analysis was performed on 87 fatty acid metabolic pathways including acetyl CoA metabolic process, fatty acid beta oxidation, and long chain fatty acid transport. Patients were categorized into high and low risk groups to compare metabolic activities based on *FAM-score*.

Table S2. CIBERSORT results.

Please refer to the files named "Table S2.xlsx"

The CIBERSORT deconvolution algorithm was performed to quantify the abundance of 22 kinds of immune cells in high-risk and low-risk subgroups.

Table S3. Gene expression matrix and *FAM-score* list of training set.

Please refer to the files named "Table S3.xlsx"

The CLL patients in the training set were grouped according to FAM-score and KEGG enrichment analysis was performed.

Table S4. Interacting genes list of CNR1, LPL, and SOCS3.

Please refer to the files named "Table S4.xlsx"

The knnDREMI (conditional-Density Resampled Estimate of Mutual Information) was applied to estimate the functional relationship of hub genes expression to other genes across the dynamic range of expression.

Supplementary figures

Figure S1

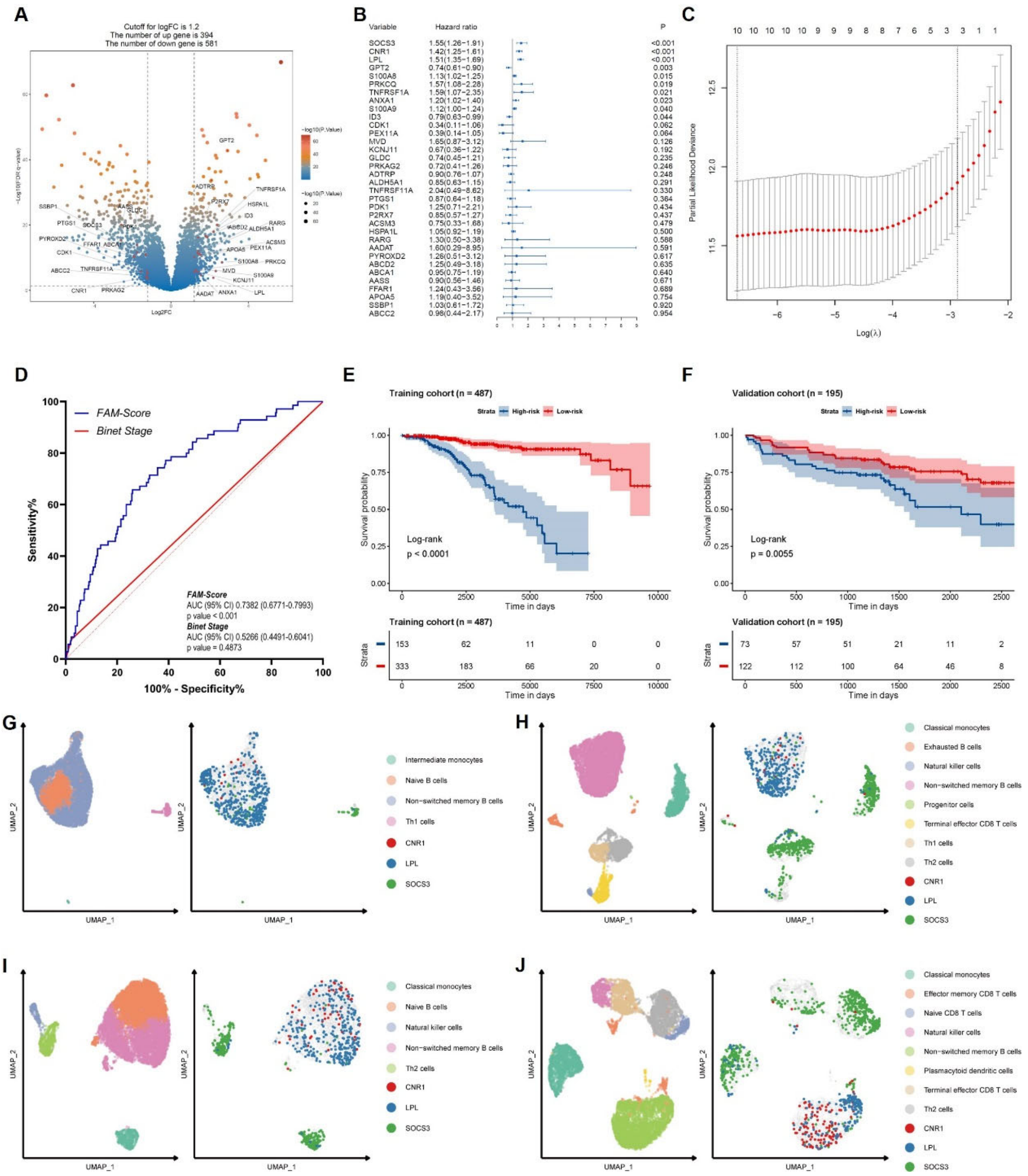

Figure S1. Construction and validation of *FAM-score*. (A) Differential expression analysis on RNA-seq data GSE50006 of CLL patients (n=180) and healthy donors (n=32). The significant difference genes related to fatty acid metabolism are marked with red dots and labels. (B) Univariate Cox regression analysis of overall survival (OS) of CLL patients on 34 genes related to FAM. (C) Cox-LASSO analysis of 10 fatty acid

metabolism-related genes significantly associated with OS. (D) ROC curve of *FAM-score* (blue) and Binet stage (red). (E-F) K-M survival curves of *FAM-score* in training set ICGC (E, n=486) and validation set GSE22762 (F, n=195). (G-J) The expression of these hub genes LPL, SOCS3, and CNR1 in CLL patients from GSE165087.

Figure S2

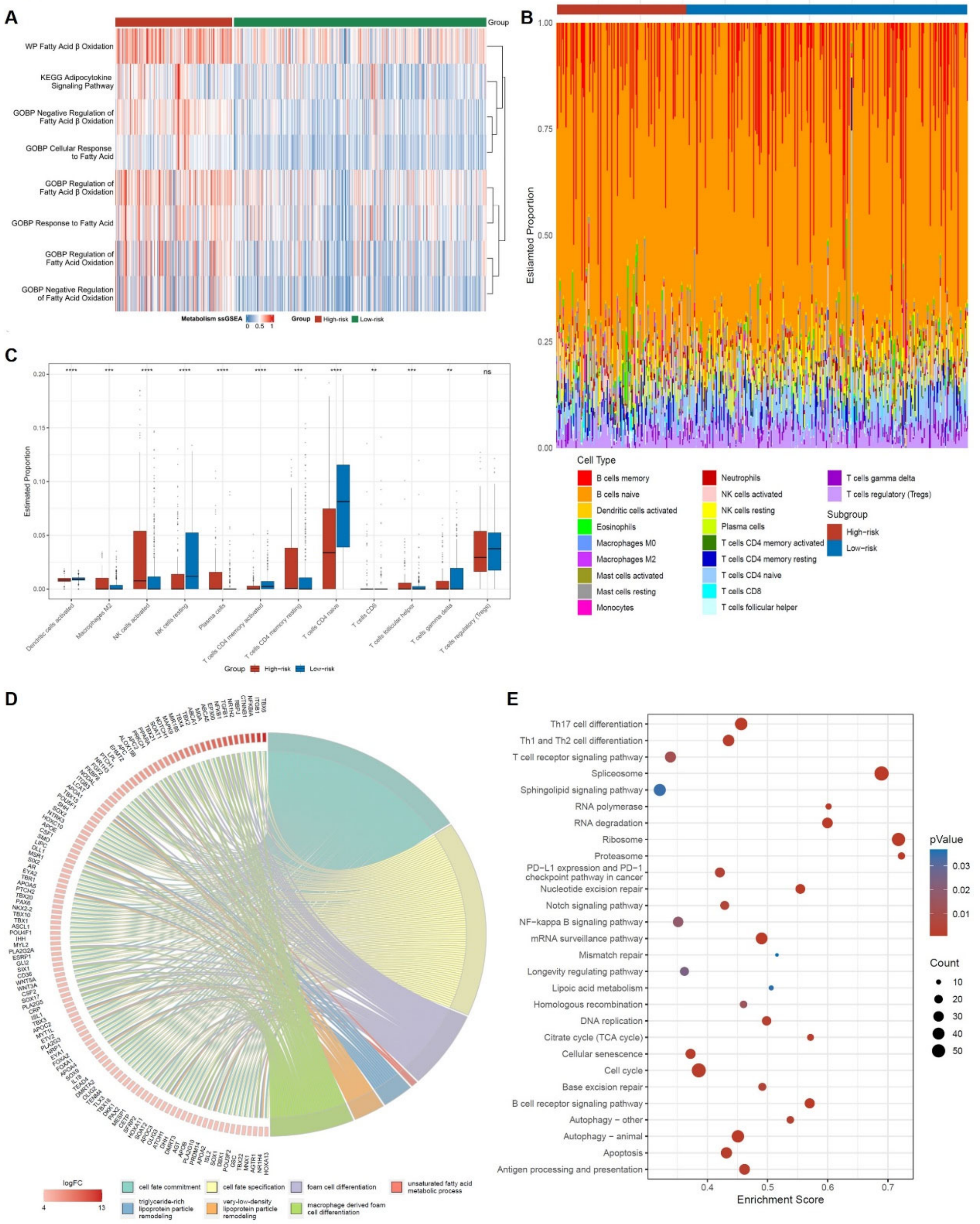

Figure S2. *FAM-score* in the role of immunophenotype regulation. (A) Comparison of FAM-related pathway activity between high- and low-risk subgroups in the ICGC cohort. Only pathways for which there were significant differences between groups are shown. See **Table S1** for more details. (B) Distribution of immune cell subpopulations in two risk subgroups in the ICGC cohort. (C) Comparison of the abundance of 22 kinds

of immune cells between high-risk and low-risk subgroups in the ICGC cohort. Symbols on the top represent significant difference. NS: not significant; *: $p < 0.05$; **: $p < 0.01$; ***: $p < 0.001$; ****: $p < 0.0001$. See **Table S2** for more details. (D) GO analysis showed that the high-risk subgroup in the ICGC cohort was enriched in cell fate commitment pathway. (E) KEGG analysis between high- and low-risk subgroups in the ICGC cohort.

Figure S3

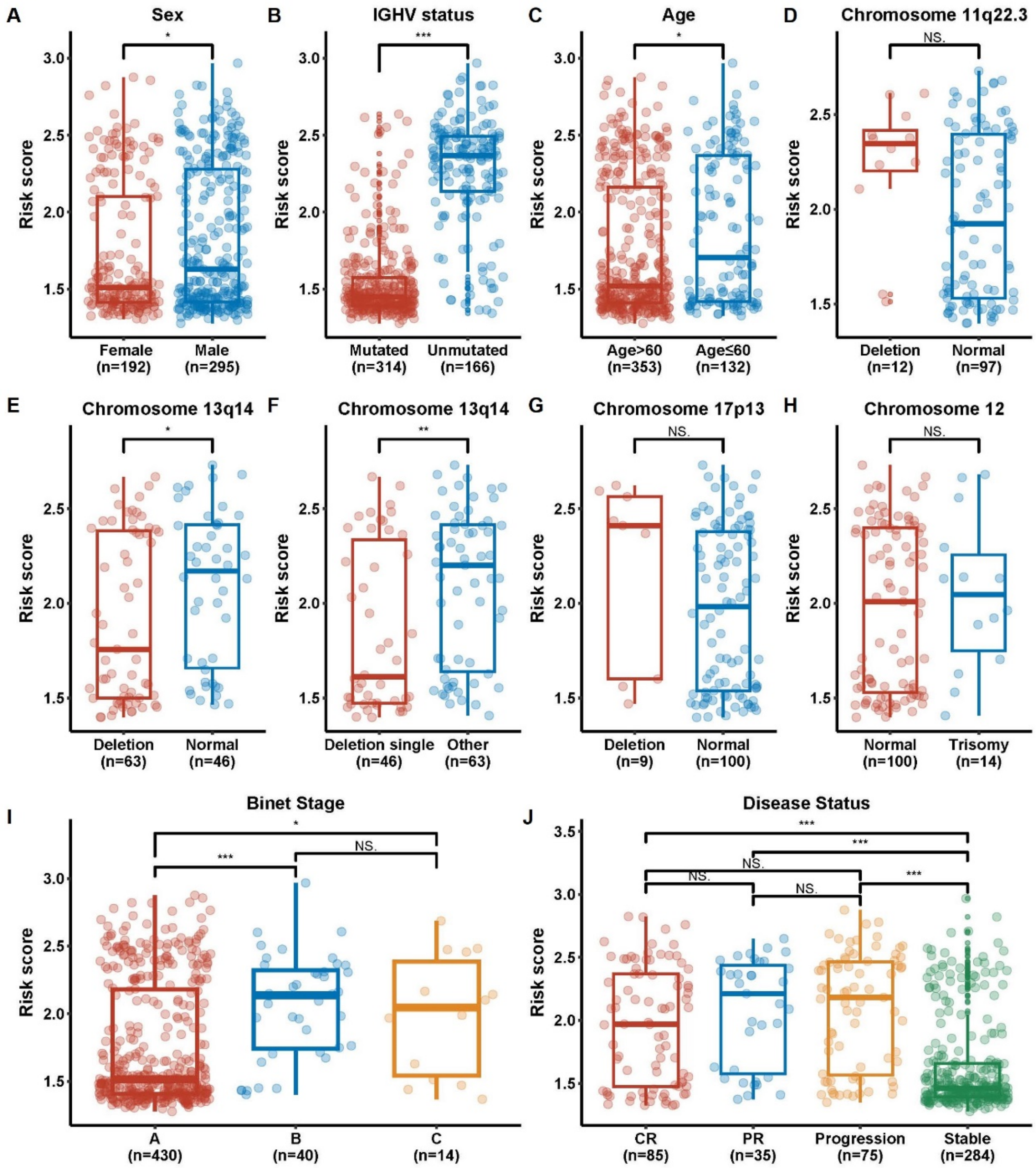

Figure S2. Altered fatty acid metabolism pathway activity between risk subgroups and *FAM-score* in the role of clinical features. (A) The *FAM-score* of male CLL patients was significantly higher than that of female CLL patients in the ICGC cohort. (B) The *FAM-score* of CLL patients without IGHV mutation was significantly higher than that of CLL patients with IGHV mutation in the ICGC cohort. (C) The *FAM-score* of elderly CLL patients was significantly lower than that of young CLL patients in the

ICGC cohort. (D) There was no significant difference in *FAM-score* between CLL patients with and without 11q22.3 deletion in the ICGC cohort. (E) The *FAM-score* of CLL patients without chromosomal alterations was significantly higher than that of CLL patients with 13q14 deletion in the GSE25571 dataset. (F) The *FAM-score* of CLL patients with other chromosomal alterations was significantly higher than that of CLL patients with 13q14 deletion in the GSE25571 dataset. (G) There was no significant difference in *FAM-score* between CLL patients with and without 17p13 deletion in the GSE25571 dataset. (H) There was no significant difference in *FAM-score* between CLL patients with and without 12 Trisomy in the GSE25571 dataset. (I) The *FAM-score* of CLL patients with Binet stage A was significantly lower than that of CLL patients with stages B and C in the ICGC cohort. (J) Inter-group comparison of *FAM-score* in CLL patients with different disease outcomes in the ICGC cohort. Symbols on the top represent significant difference. NS: not significant; *: $p < 0.05$; **: $p < 0.01$; ***: $p < 0.001$.

Figure S4

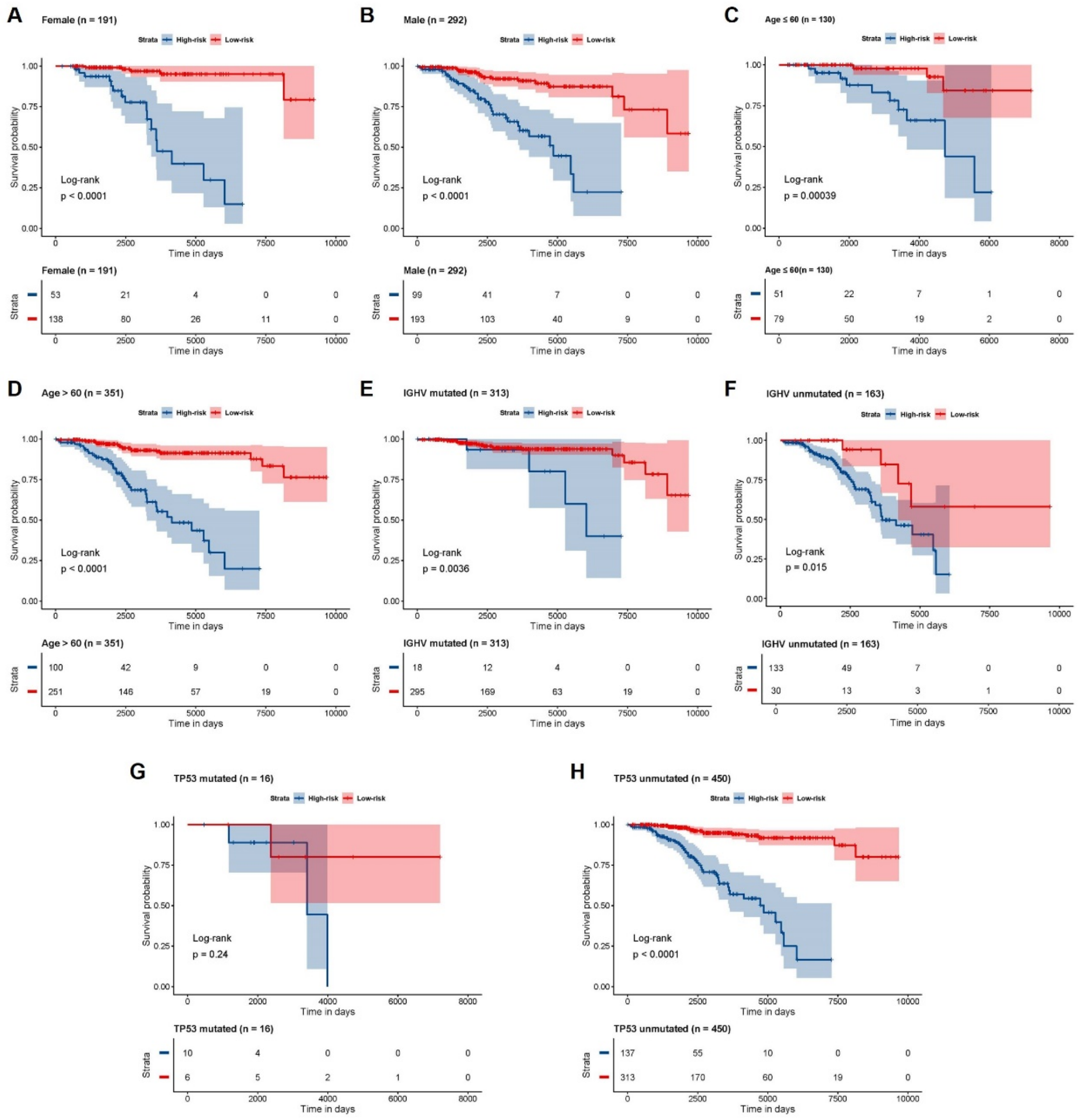

Figure S3. Kaplan-Meier analysis in CLL patients with different clinical characteristics. (A-B) The Kaplan-Meier curves of *FAM-score* in CLL patients in the ICGC cohort of different sexes. (C-D) The Kaplan-Meier curves of *FAM-score* in CLL patients in the ICGC cohort of different ages. (E-F) The Kaplan-Meier curves of *FAM-score* in CLL patients in the ICGC cohort of different IGHV mutation status. (G-H) The Kaplan-Meier curves of *FAM-score* in CLL patients in the ICGC cohort of different TP53 mutation status.
